# Editorial: Translational research on neuropathic pain and headache

**DOI:** 10.3389/fneur.2022.1024033

**Published:** 2022-09-08

**Authors:** Bamidele Victor Owoyele, Roi Treister, Télesphore Benoît Nguelefack, Daniel Ciampi De Andrade

**Affiliations:** ^1^Neuroscience and Inflammation Unit, Department of Physiology, Faculty of Basic Medical Sciences, College of Medicine, University of Ilorin, Ilorin, Nigeria; ^2^Department of Nursing, Faculty of Social Welfare and Health Sciences, University of Haifa, Haifa, Israel; ^3^Research Unit of Neuro-inflammatory and Cardiovascular Pharmacology, Department of Animal Biology, Faculty of Science, University of Dschang, Dschang, Cameroon; ^4^Department of Neurology, School of Medicine, University of São Paulo, São Paulo, Brazil

**Keywords:** animals, headache, migraine, neuropathic pain, translational research

Pain is the most common factor that drives hospital and healthcare facility visits by patients (1–3). It has also been estimated that 1 out of 5 adults suffer pain worldwide and 10% of adults are diagnosed with at least one form of chronic pain (4). Among the different types of chronic pain, neuropathic pain, and headache are considered to be the most intractable (5, 6). Neuropathic pain formerly known as “pain initiated or caused by a primary lesion, dysfunction or transitory perturbation of the peripheral or central nervous system” has been reshaped by the International Association for the Study of Pain as “pain caused by a lesion or disease of the somatosensory nervous system” (7). The number of patients with neuropathic pain is increasing astronomically worldwide probably due to causes such as exposure to environmental toxins, drugs, metabolic derangements, infections, and surgeries (8). A headache is also a form of pain with high incident rates and different causes. Headache disorders have been reported to be among the most prevalent and disabling ailments globally (9). The morbidity caused by headache and neuropathic pain has significant effects on labor and productivity as precious hours are lost when individuals and workers are incapacitated by these conditions (8–10). Besides, these pathologies drastically affect the quality of life since patients usually undergo episodic and recurring manifestations. Because of the complexity of the two diseases, their classifications have been a challenge and their physiopathology is poorly understood, despite the large amount of related physiological and biochemical research works. Headache especially migraine is regarded as part of neuropathic pain. In fact, migraine is considered a neurogenic disorder associated with secondary changes in brain perfusion (11–13).

With such limits and ambiguity in the understanding of the physiopathology and defining neuropathic pain and headache, the management of the diseases becomes difficult. In addition, as the two pathologies are resistant to the existing drugs, new strategies and alternatives are necessary to overcome the therapeutic difficulties (14, 15). Thus, translational research should address these gaps to better the quality of life of neuropathic pain and headache patients.

The aim of this Research Topic was to gather information on neuropathic pain and headache, which both lead to chronic pain. It was also envisaged that manuscripts published under this Research Topic would shed light on new insights into the physiopathology and on novel treatments for both types of pain.

Concerning the physiopathology, Lin et al. showed that the tension of the cervical extensor muscle contributes to the cervicogenic headache. This is supported by the fact that stiffness in the superficial extensor muscles was significantly higher on the side of the headache than on the contralateral side. More, there was a significantly higher tension in the unaffected side of the patients than in the control subjects. The stiffness of semispinalis cervicis was positively correlated with VAS scores in cervicogenic headache patients. Li et al. shed light on the probable mechanisms underlying chronic pain due to Postherpetic Neuralgia (PHN), which arises as a complication of herpes zoster. The experimental study in mice showed that the injection of Varicella-Zooster Virus (VZV) to the footpads of mice led to upregulation of T-Type calcium channel Cav 3.2 expression in the dorsal root ganglia and spinal dorsal horn of these mice. The injection of Cav.3.2 channel blocker (2R/S)-6-prenylnaringenin relieved the pain associated with VZV injection. The findings showed that Cav. 3.2 channel could be a putative therapeutic channel for the treatment of PHN.

Yang et al., provided insight into the identification of possible blood biomarkers for all types of migraine and the critical role that oxidative stress might be playing in the pathogenesis of the disease. The findings from their study highlighted the potential role of serum levels of Uric Acid (UA), Total bilirubin (TBil), Albumin (ALB), and Creatinine (CRE) in migraine. Specifically, ALB, TBil, and UA were independently related to migraine. They concluded that oxidative stress is a factor to be considered in the etiology and pathogenesis of migraine.

Some of the papers published in this special topic highlight new therapeutic and alternative approaches. In fact, Anand et al. exhibited the beneficial effects of capsaicin 8% patch for the treatment of Non-Freezing Cold Injury. Their findings showed that capsaicin 8% patch reduced spontaneously evoked pain. This interesting therapeutic approach not only alleviates neuropathic pain but is able to induce nerve regeneration and restoration. Malfitano et al. reported a case study involving a patient with thalamic stroke presenting with acute onset pain and paresthesia. The observations showed that the patient responded well to repetitive transcranial magnetic stimulation (rTMS) applied to the hand area of the primary motor cortex. The significantly ameliorated pain was also accompanied by a decreased motor cortex excitability. The findings represent the first report on the use of rTMS for the treatment of acute central post-stroke pain.

In conclusion, this Research Topic has collated both basic and clinical research. The findings of the published articles are of good translational value but call upon additional works on both the physiopathology pathways and therapeutical approaches to neuropathic pain and headache.

## Author contributions

BO produced the first draft. RT, TN, and DA revised the draft. All authors approved the submission.

## Funding

BO is supported by Tertiary Education Trust Fund (TETFund) of the Federal Government of Nigeria under Grant No. TETFUND/DESS/NRF/STI/11/Vol.1. DA is supported by a Novo Nordisk Grant (NNF21OC0072828).

## Conflict of interest

The authors declare that the research was conducted in the absence of any commercial or financial relationships that could be construed as a potential conflict of interest.

## Publisher's note

All claims expressed in this article are solely those of the authors and do not necessarily represent those of their affiliated organizations, or those of the publisher, the editors and the reviewers. Any product that may be evaluated in this article, or claim that may be made by its manufacturer, is not guaranteed or endorsed by the publisher.
